# Misinformation About the Human Gut Microbiome in YouTube Videos: Cross-sectional Study

**DOI:** 10.2196/37546

**Published:** 2022-05-16

**Authors:** Swathikan Chidambaram, Yathukulan Maheswaran, Calvin Chan, Lydia Hanna, Hutan Ashrafian, Sheraz R Markar, Viknesh Sounderajah, John C Alverdy, Ara Darzi

**Affiliations:** 1 Department of Surgery & Cancer Imperial College London London United Kingdom; 2 Institute of Global Health Innovation Imperial College London London United Kingdom; 3 Department of Molecular Medicine and Surgery Karolinska Institutet Stockholm Sweden; 4 Department of Upper Gastrointestinal Surgery Churchill Hospital Oxford University Hospitals National Health Service Trust Oxford United Kingdom; 5 Department of Surgery University of Chicago Chicago, IL United States

**Keywords:** microbiome, social media, YouTube, misinformation, content analysis, gut health, misinformation, public

## Abstract

**Background:**

Social media platforms such as YouTube are integral tools for disseminating information about health and wellness to the public. However, anecdotal reports have cited that the human gut microbiome has been a particular focus of dubious, misleading, and, on occasion, harmful media content. Despite these claims, there have been no published studies investigating this phenomenon within popular social media platforms.

**Objective:**

The aim of this study is to (1) evaluate the accuracy and reliability of the content in YouTube videos related to the human gut microbiome and (2) investigate the correlation between content engagement metrics and video quality, as defined by validated criteria.

**Methods:**

In this cross-sectional study, videos about the human gut microbiome were searched for on the United Kingdom version of YouTube on September 20, 2021. The 600 most-viewed videos were extracted and screened for relevance. The contents and characteristics of the videos were extracted and independently rated using the DISCERN quality criteria by 2 researchers.

**Results:**

Overall, 319 videos accounting for 62,354,628 views were included. Of the 319 videos, 73.4% (n=234) were produced in North America and 78.7% (n=251) were uploaded between 2019 and 2021. A total of 41.1% (131/319) of videos were produced by nonprofit organizations. Of the videos, 16.3% (52/319) included an advertisement for a product or promoted a health-related intervention for financial purposes. Videos by nonmedical education creators had the highest total and preferred viewership. Daily viewership was the highest for videos by internet media sources. The average DISCERN and Health on the Net Foundation Code of Conduct scores were 49.5 (SE 0.68) out of 80 and 5.05 (SE 2.52) out of 8, respectively. DISCERN scores for videos by medical professionals (mean 53.2, SE 0.17) were significantly higher than for videos by independent content creators (mean 39.1, SE 5.58; *P*<.001). Videos including promotional materials had significantly lower DISCERN scores than videos without any advertisements or product promotion (*P*<.001). There was no correlation between DISCERN scores and total viewership, daily viewership, or preferred viewership (number of likes).

**Conclusions:**

The overall quality and reliability of information about the human gut microbiome on YouTube is generally poor. Moreover, there was no correlation between the quality of a video and the level of public engagement. The significant disconnect between reliable sources of information and the public suggests that there is an immediate need for cross-sector initiatives to safeguard vulnerable viewers from the potentially harmful effects of misinformation.

## Introduction

Over the past decade, governments, health organizations, and private sector companies have increasingly endorsed the use of social media platforms as a trusted means of disseminating information on health and wellness [1-3]. Of the available platforms, YouTube has arguably emerged as the standout modality for accessing comprehensive health care information. As of December 2021, YouTube boasts over 1 billion hours of watched content daily, over 2 billion unique users monthly, and is commonly accessed by both health professionals and the public alike for a wide range of health-related queries [4]. Although YouTube usually hosts factually accurate content, there have been reported instances where inaccurate information has been hosted for public viewing [5]. This issue came to prominence during the COVID-19 pandemic as inaccurate content about COVID-19 vaccines and national vaccination programs rapidly gained global viewership. This, in turn, led to YouTube’s implementation of a COVID-19 medical misinformation policy, which disallowed COVID-19 content that contradicts the policies of health authorities [6]. Since its implementation in September 2020, the platform has removed over 130,000 videos, a staggering figure highlighting the scale of misinformation on vital public health matters. However, a similarly specific policy has not been extended to other areas of health-related content in which there may be a history of misinformed content. In these cases, the responsibility of determining the veracity and applicability of the encountered information is placed upon the individual [7].

As increasing understanding develops around the “digital determinants of health” [8], there is concern that unverified content may lead to instances of clinical harm to viewers who cannot independently discern content quality [9-11]. In turn, these users may share misinformed content with their social networks, resulting in misinformation within epistemic bubbles and, more concerningly, “echo chambers” [12,13]. One topic that is highly vulnerable to this phenomenon is the human gut microbiome. The gut microbiome is a consistently popular topic for content creators on YouTube, particularly as greater understanding develops around its regulatory role in anxiety, mood, cognition, and pain through the gut-brain axis. Despite the paucity of high-grade evidence on the mechanisms through which the microbiome may be reconfigured to optimize human physiology, there has been a sharp rise in videos that promote lifestyle-based strategies which purport to modify the gut microbiome for health and well-being benefits [14,15]. Without rigorous standards related to scientific accuracy, these videos often stretch the applications of existing scientific evidence to advocate treatments that are advertised as helpful but instead may have no effect or potentially cause overall harm. Hence, if not properly caveated with scientific resources and disclaimers, these videos can distract viewers from empirically sourced medical advice and protective health-seeking behaviors.

To date, no studies have evaluated the quality of the microbiome-related content available on social media. Moreover, it is not known whether the engagement metrics associated with microbiome-related content correlate with the quality of the information provided. As such, this study aims to (1) evaluate the accuracy and reliability of YouTube content pertaining to the human gut microbiome and (2) investigate the correlation between social media engagement metrics and video quality, as defined by validated criteria.

## Methods

### Ethical Considerations

The institutional review board of Imperial College London determined this study does not constitute human subjects research and was therefore exempt from the associated ethical requirements.

### Selection of Videos

The phrases “microbiome”, “gut microbiome”, and “microbiome health” were searched on the United Kingdom (UK) version of YouTube on September 20, 2021. The search terms were chosen to ensure appropriate coverage of any videos related to the role of the gastrointestinal microbiome in health and well-being. The search was conducted using an incognito browser (Google Chrome, Google LLC) to avoid biased suggestions based on cookies or previous search histories. The results were ranked according to view count as this is the most sensitive means of identifying videos that have had the greatest impact and are the most likely to trend. The 200 most-viewed videos (10 pages) of each search were deduplicated and subsequently extracted. Video titles and channels were first screened for relevance and English language before further full-video screening. Videos were included if they described at least 1 of the following: (1) components of the gut microbiome; (2) the role of the microbiome in gut health, including cancers, inflammatory bowel conditions, and infectious bowel conditions; (3) methods of altering the gut microbiome for the purpose of broader health-related effects; and (4) side effects or the safety of interventions used. Videos that did not discuss the human gut microbiome or were not in the English language were not included. Videos >5 years old were also excluded to represent the current body of work about the microbiome more accurately. In cases of uncertainty on whether a video should be included, a consensus was sought between authors, with a tendency to include the video for full assessment.

### Data Extraction

The characteristics of the videos were independently extracted by 2 authors (SC and YM). This included the video’s URL, country of origin, duration, age, and channel name. Engagement metrics that assess the use of a video by the public were also extracted; this included the number of likes, dislikes, comments, and the view count. The videos were subsequently classified into the following 6 categories based on their content and purpose: educational channels produced by medical professionals (eg, the American Society for Microbiology); educational channels produced by nonmedical professionals (science education or explanatory media, eg, The Sheekey Science Show), independent producers (eg, Tom Bilyeu – QUEST nutrition), internet media (magazines or talk shows, eg, Health Via Modern Nutrition), news agencies (clips from network news programs, eg, King 5 news), and nonprofit or medical organizations (hospitals, government organizations, or universities eg, Mayo Clinic, UCLA Health, Hopkins Kimmel).

### Outcome Measures

The primary outcome measure was the quality of the videos assessed, which is graded using the DISCERN tool as well as by assessing the video’s adherence to the Health on the Net Code of Conduct (HONcode). Secondary measures included engagement metrics, which were classified as total viewership (total number of views the video accrued), daily viewership, and preferred viewership (number of likes received by the video). Correlations between video characteristics, engagement metrics, and quality assessment scores were analyzed using linear regression models.

### The DISCERN Score

The DISCERN tool is an instrument that is designed to help users assess consumer health information. The criteria were originally drafted based on the analysis of a random sample of consumer health information on the treatment choices for 3 medical conditions (myocardial infarction, endometriosis, and chronic fatigue syndrome). The tool consists of 3 categories of items, scored between 1 (worst) and 5 (best), that assess the reliability (8 questions, 40 attainable points), quality of Information (7 questions, 35 attainable points), and overall impression (1 question, 5 attainable points) of the information source, thus accounting for a total attainable score of 80. The DISCERN criteria have enabled the assessment of various aspects of video quality, including completeness, understandability, relevance, depth, and accuracy of information. The shortlisted videos were rated independently by 2 authors (SC and YM). Any discrepancies were resolved through discussion with a third author (VS).

### The HONcode

The HONcode certification is an ethical standard aimed at offering high-quality health information. It demonstrates the intent of a website to publish reliable and high-quality information with maximum transparency. The HONcode consists of 8 principles that evaluate the reliability and credibility of health information, including the justification for and balance of claims, citations of sources used, details of funding, and clear distinguishment of advertising from editorial content [16]. Videos were rated with a score of 1 (adherent) or 0 (nonadherent) for each of the 8 principles, for a total score of 8.

### Statistical Analysis

The statistical analysis was performed using SPSS (version 27; IBM Corp). All data are presented as means and SEs of means unless otherwise stated. The interrater reliability was assessed using the Cohen κ statistic. A DISCERN score of 1 or –1 was considered agreement for each category in accordance with previous studies. Data were assessed for normality using the Shapiro-Wilk test. The 1-way ANOVA and Kruskal-Wallis tests were used to assess the relationships between categorical variables, such as channel type, and DISCERN scores with parametric and nonparametric distributions, respectively. The associations between engagement metrics and DISCERN scores were evaluated using linear regression. Statistical significance was set at *P*<.05.

## Results

### Video Selection and Characteristics

**Figure 1:**
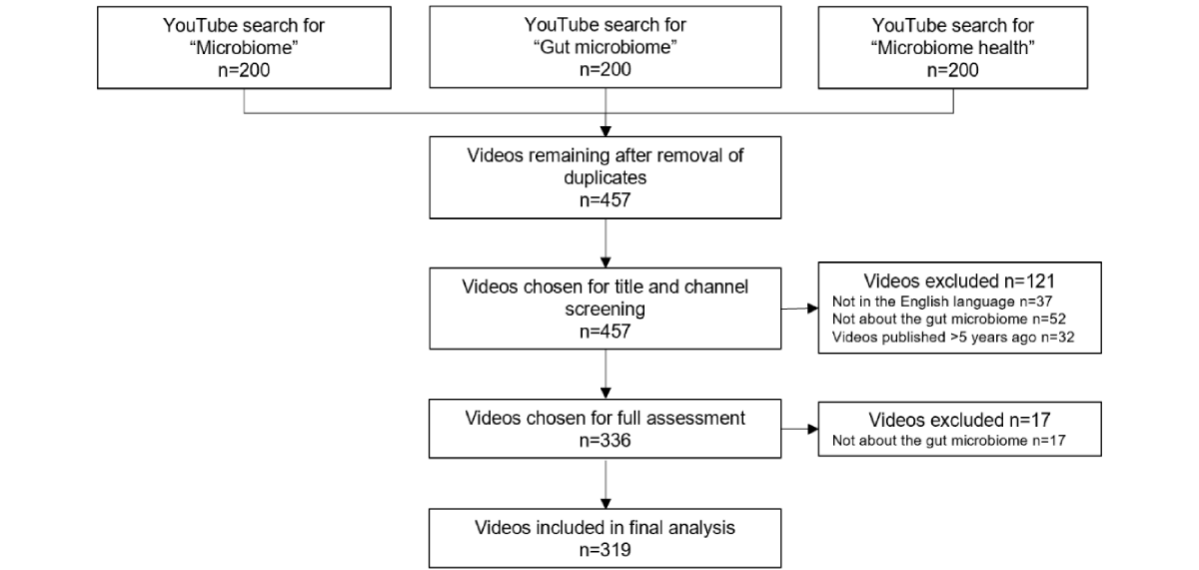
The results of searches for microbiome-related videos on YouTube and the video selection process for inclusion in the study.

We identified 600 videos in total based on the 3 search terms. Following deduplication, 143 videos were removed, yielding 457 videos. After the initial screening of video titles and channels, 138 videos that did not meet the inclusion criteria were removed. This led to 319 videos being included in the final analysis. The video review process is illustrated in Figure 1.

Of the 319 videos included in this study, the majority originated from North America (234/319, 73.4%), followed by Europe (46/319, 14.4%) and Australasia (32/319, 10%). A total of 41.1% (131/319) of the videos were produced by nonprofit organizations, 15.4% (n=49) were created by independent creators, and 11.9% (n=38) were produced by nonmedical educational organizations. In total, only 19.4% (62/319) of the videos were produced by a medical professional, of which a smaller minority had proven expertise in the human microbiome. The mean duration of videos was 21.2 (SE 1.25) minutes. A majority (251/319, 78.7%) of the videos were uploaded in the last 3 years, with a median age of 2.22 years. Of note, 16.3% (52/319) of the videos included an advertisement for a product or promoted a health-related intervention for financial purposes. The characteristics of the included videos are summarized in Table 1.

**Table 1:** Characteristics of the sample videos (N=319).

Characteristic		Value
**Country of origin, n (%)**		
	Australia	28 (8.8)
	Canada	10 (3.1)
	France	2 (0.6)
	Germany	3 (0.9)
	Hungary	1 (0.3)
	India	3 (0.9)
	Ireland	3 (0.9)
	Italy	1 (0.3)
	Lebanon	1 (0.3)
	The Netherlands	1 (0.3)
	New Zealand	4 (1.3)
	Russia	1 (0.3)
	South Africa	3 (0.9)
	Spain	1 (0.3)
	Switzerland	1 (0.3)
	United Kingdom	32 (10)
	United States of America	224 (70.2)
**Channel type, n (%)**		
	Educational (nonmedical)	38 (11.9)
	Educational (medical)	62 (19.4)
	Independent users	49 (15.4)
	Internet media	32 (10)
	News agency	7 (2.2)
	Nonprofit	131 (41.1)
Duration in minutes, mean (SE)		21.2 (1.25)
Days since upload, mean (SE)		962 (40.1)
**Engagement**		
	Views, mean (SE)	195,469 (43,985)
	Views per day since upload, mean (SE)	245 (47.8)
	Likes, mean (SE)	3954 (909)
	Dislikes, mean (SE)	90.8 (21.2)
Containing advertisements or serving a promotional purpose, n (%)		52 (16.3)

### Quality Assessment Scores

**Figure 2:**
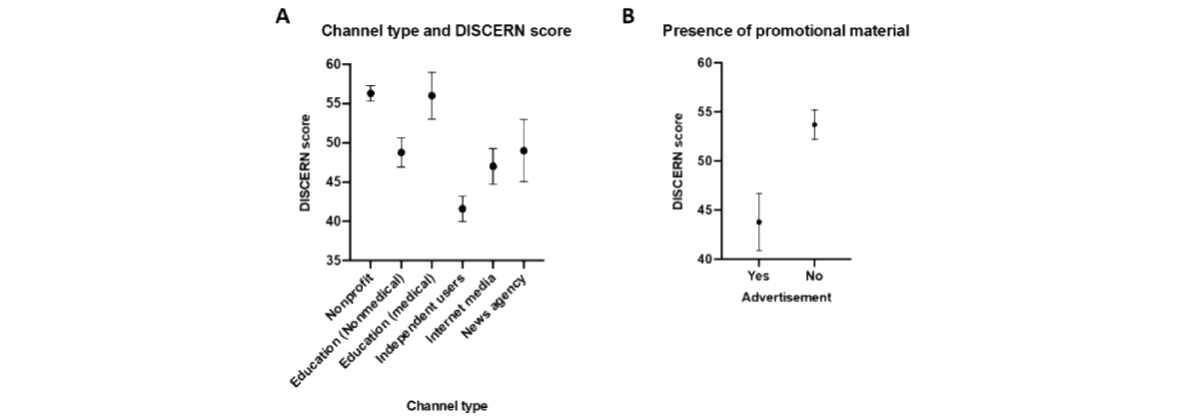
The relationships between video characteristics and DISCERN scores. All values are presented as averages with 95% CIs.

The average DISCERN and HONcode scores were 49.5 (SE 0.68) out of 80 and 5.05 (SE 2.52) out of 8, respectively. Of the various video characteristics, the regression analysis shows that only the channel type and the presence of promotional materials were indicative of content quality, as per DISCERN scoring (Figure 2). Figure 2A shows the effect of the channel type on the mean DISCERN score, and Figure 2B shows the effect of promotional materials on the mean DISCERN score. Videos with promotional materials had significantly lower DISCERN scores than videos without any advertisements or product promotion (*P*<.001). The highest DISCERN scores were recorded for videos by medical professionals (mean 53.2, SE 0.17), whereas lower DISCERN scores were recorded for videos by independent content creators (mean 39.1, SE 5.58). Scores for each of the DISCERN subsections (completeness, understandability, relevance, depth, and accuracy of information) followed a similar trend.

**Figure 3:**
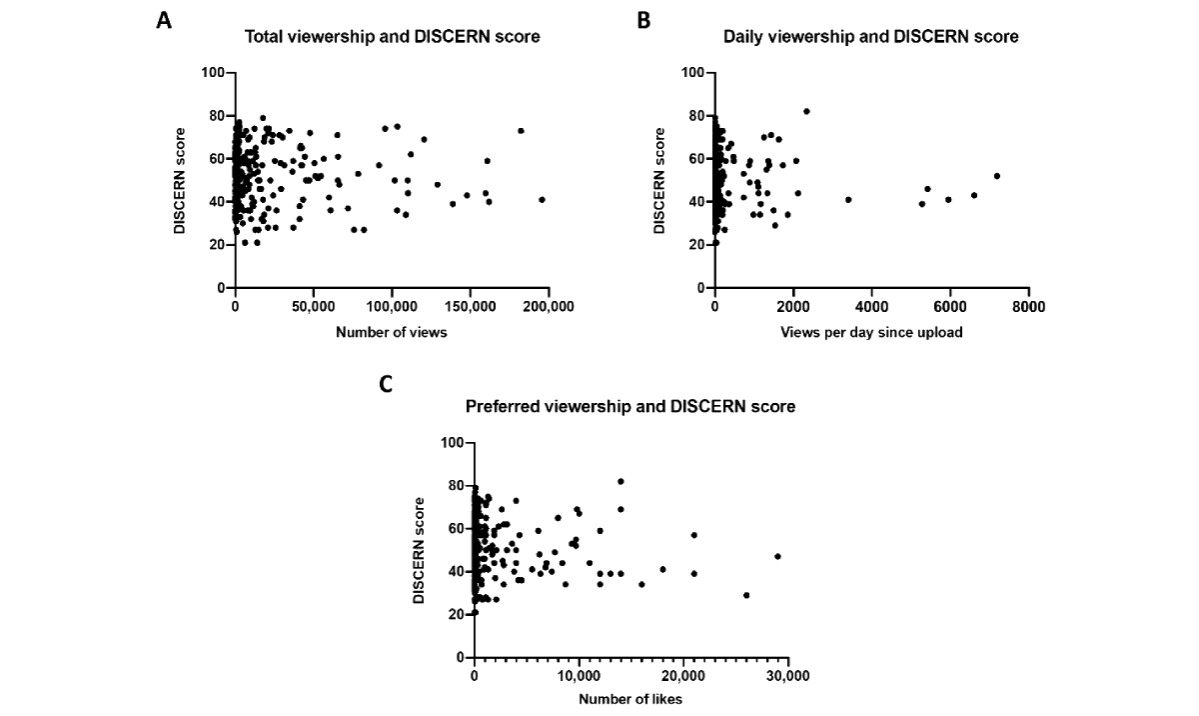
The relationship between engagement metrics and DISCERN scores, highlighting the correlation between the total number of views (A), daily viewership (B), and number of likes (C).

The linear regression analysis revealed no correlation between the quality of a video based on the DISCERN score and any engagement metric, including total viewership, daily viewership, and preferred viewership (number of likes; Figure 3). There was some agreement between the HONcode and DISCERN scores. For example, the HONcode score was the highest for videos by medical professionals, which also had the highest DISCERN scores. However, this was not a consistent correlation as HONcode scores were significantly lower (*P*<.001) for videos by internet media sources, at a mean of 1.28 (SE 0.81), relative to the mean DISCERN score of 47.7 (SE 4.18).

### Engagement Metrics

Overall, the videos accrued a total of 62,354,628 views since being uploaded. The highest mean number of views was achieved by nonmedical education creators. The viewership per video for content produced by medical professionals was 136,606, which was significantly lower than the average of 195,469 views for all videos. Preferred viewership followed a similar trend, in which the highest value of 10,920 likes was registered for videos made by nonmedical education creators; this was 3 times the value for videos by health care professionals (n=3480) and 5 times the value for videos from nonprofit organizations (n=2954). In contrast, daily viewership (n=1067) was the highest for internet media sources and was thrice that achieved by medical professionals (n=321). In parallel, videos by internet media sources were liked (n=6020) to a greater extent relative to total viewership and compared to other channel types, reflecting greater approval among the audience. Summary data of engagement metrics are included in Table 2.

**Table 2:** Engagement metrics, adherence to the Health on the Net Foundation Code of Conduct, and DISCERN scores for YouTube videos on the gut microbiome.

Parameter	Value for each channel type							Value for all channel types
	Educational (nonmedical)	Educational (medical)	Independent nonmedical users	Internet media	News agencies	Nonprofit organizations		
Videos, n	38	62	49	32	7	131	319	
Total views, n	16,786,496	8,469,633	5,909,797	3,014,580	727,969	27,446,153	62,354,628	
Views per video, n	441,749	136,606	120,608	94,205	103,995	209,512	1,106,675	
Daily views, n	436	321	234	1067	40	156	2254	
Likes, n	10,920	3480	3196	6020	1162	2954	27,732	
Dislikes, n	228	31	54	245	17	98	673	
HONcode adherence score (out of 8), mean (SE)	2.36 (0.16)	2.77 (0.8)	2.48 (0.35)	2.03 (0.17)	1.28 (0.81)	2.67 (0.13)	5.05 (2.52)	
DISCERN total score (out of 80), mean (SE)	46.4 (1.85)	53.2 (0.17)	39.1 (5.58)	44.9 (2.25)	47.7 (4.18)	53.6 (0.91)	49.5 (0.68)	
DISCERN reliability score (out of 40), mean (SE)	24.6 (0.96)	27.4 (0.8)	18.5 (2.65)	22.6 (1.23)	22.6 (2.29)	27.6 (0.47)	25.2 (0.38)	
DISCERN quality score (out of 35), mean (SE)	18.9 (0.91)	22.3 (0.63)	18.1 (2.58)	19.6 (1.02)	21.7 (0.35)	22.4 (0.45)	21.0 (0.31)	
DISCERN overall impression score (out of 5), mean (SE)	2.84 (0.1)	3.51 (0.09)	2.51 (0.35)	2.75 (0.16)	3.42 (0.35)	3.61 (0.06)	3.24 (0.05)	

## Discussion

### Principal Findings

This study highlights the importance of YouTube as a medium for sharing content surrounding the human gut microbiome. We demonstrate increasing public interest in this field, with over 62 million cumulative views on the videos that were shortlisted. However, this study also highlights that there is significant variation in the quality of the information provided by most videos. A content analysis revealed that the quality of the information was dependent on factors such as the profile of the content creator and the presence of a financial slant. Interestingly, videos with the most reliable information did not consistently receive the highest ratings or engagement. The disconnect between content quality and public engagement suggests that less informed videos are being viewed in lieu of more balanced content, which could lead to the propagation of misinformation.

Our analysis showed an inverse relationship between channel type, quality of content, and viewership. Videos created by recognized institutions, such as universities, hospitals, and charities, provided more accurate information than videos from independent producers, some of whom were also qualified medical practitioners. However, both the total and average viewership was significantly higher for nonmedical content creators, even after accounting for the duration of the videos and date of upload. The number of likes given to videos reflected a similar pattern, indicating that viewers expressed a more positive reception of videos that were lower in quality. This highlights the alarming phenomenon that low-quality, unreliable, and potentially misinformed content may be more readily acquired, processed, and positively viewed by an audience who may otherwise be unaware of existing literature on the microbiome [17].

Furthermore, the majority of videos made by independent creators captured in this study usually promoted a specific form of health care intervention, such as a dietary product, to “reengineer” the microbiome [18]. Although the microbiome inarguably plays a crucial role in a wide range of common medical conditions, there is a paucity of high-grade evidence demonstrating a risk modification effect of a reconfigured microbiome or that the advertised interventions can modify the microbiome to a clinically significant extent. These videos, often with no declaration of financial interests, frequently lacked evidence or misrepresented the literature by making a significant leap in the interpretation of the results of existing literature on the microbiome. Thus, given the far-reaching effects of web-based misinformation, there is a pressing need for key stakeholders, such as content creators, governments, health organizations, and hosting platforms, to proactively implement policy, regulatory, and educational interventions to protect susceptible members of the population.

### Policy Implications

Particularly in the postpandemic era, digital technologies are increasingly commonplace in the delivery of public health and well-being educational materials and interventions. Although younger cohorts may have embraced this shift, there is evidence to suggest that many segments of the population were poorly prepared for this rapid digitization of services [19]. Equity across the population in the ability to access, interpret, and appraise information is both under-reported and overlooked. Targeted interventions need to empower the end-user as well as serve a custodial role over the production of content. As such, strategies to mitigate the spread of nonfactual health information on social media can be approached at 3 levels: government, industry, and consumer.

At the government level, there is laudable tightening in protective legislation globally. The proposed UK legislation (the Online Safety Bill) aims to combat harmful web-based content through increased regulatory oversight [20]. Similarly in the United States, the proposed Health Misinformation Act and Justice Against Malicious Algorithms Act aim to hold web-based platforms accountable for posting content with misinformation related to an existing public health emergency (eg, COVID-19) or contributing to physical or severe emotional injury [21-23].

Additionally, in the context of COVID-19 and the spread of anti-vaccination misinformation, technology corporations have already begun to remove and monitor nonfactual and harmful content on their sites. YouTube’s search algorithm has historically recommended videos that attracted the most views or clicks. However, the recent heightened concerns regarding harmful misinformation on YouTube have prompted algorithm changes, which have reportedly reduced the consumption of borderline content by 70% [24]. Further potential action through increasing the ranking and visibility of health content from reputable scientific sources, such as universities, hospitals and health charities, would increase consumer exposure to high-quality and reliable health information. To appraise content, platforms need to generate criteria for evaluating the credibility and reliability of a source; evaluate which assessment tools such as the HONcode, URAC certification or the Credibility, Reliability, Authority, and Purpose test best fit their model; and incorporate them into their quality appraisal methods. As part of this, platforms can also necessitate that content creators disclose potential conflicts of interest to minimize instances where content is skewed for the purpose of commercialization. The ground layout (ie, content guidelines) where a social media platform has outlined clear criteria for credibility and ways to achieve it will be useful for content creators to follow when producing their content. In this way, social media platforms can actively elevate high-quality content while reducing misinformation from poorer-quality content. A summary of recommendations for content-hosting platforms is displayed below (Textbox 1).

**A summary of recommendations for content-hosting platforms to increase the quality and reliability of visible health-related content.**

- Evaluate content with assessment tools (eg, Health on the Net Foundation Code of Conduct, URAC, or Credibility, Reliability, Authority, and Purpose test).
- Increase ranking, and therefore visibility, of health content from reputable scientific sources.
- Necessitate disclosure of conflicts of interest from content creators.
- Flag content from nonmedical independent creators with content warnings.
- Provide external links to health content from validated sources of health information (eg, United Kingdom National Health Service, World Health Organization, US Centers for Disease Control and Prevention).
- Outline clear criteria for content creators to achieve increased credibility.

Consumers also have a responsibility to identify nonfactual information and discern high-quality and reliable information on the internet. Often, social media sites allow users to tailor their preferences and see information from only the sources they select, leading to “bubbles” and echo chambers that reinforce any false information users encounter. Given the vast amount of content that is uploaded to the internet on a continuous basis, it is unrealistic for content hosts to review all material for nonfactual or harmful content. Thus, the provision of educational material for the general public is crucial, especially given that health information resources are increasingly being migrated to the internet. Globally, the World Health Organization, in partnership with local governmental agencies, has introduced several initiatives to improve public awareness and education regarding web-based health misinformation, with a particular focus on COVID-19 and vaccination misinformation [25,26]. Additionally, the UK government has recently published its Online Media Literacy Strategy, with one of the key aims being to improve the ability of members of the public to identify misinformation and assess the reliability of a web-based information source [27]. Similar to likes and ratings, social media platforms can employ verified users who have prior experience in using validated tools to assess video quality to provide their feedback. Videos identified can be checked by the platform before consideration for removal as well. It is important that the crude censorship of these videos does not foray into impeding free speech and still allows the user at the heart of the consumption process to make the final decision. With an increasing reliance on digital health technology that is only expected to rise in the future, it is critical to ensure the public is adequately equipped with the skills to use it, and the ability to recognize and manage misinformation forms a significant component of this.

### Strengths and Limitations

This study has several strengths. Firstly, to our knowledge, this is the first study to report on the accuracy and quality of the most widely viewed YouTube videos about the gut microbiome. Furthermore, we have analyzed the videos’ content accuracy and reliability using a combination of objective measures, including the DISCERN criteria. Our sample size is extensive and covers up to 400 of the most-watched videos on this topic, capturing over 62 million views. As such, our study provides a broad assessment of the mismatch between content quality and viewership, which provides important insights into channels through which key stakeholders may codesign interventions to deliver high-quality information to an evidently receptive audience. Limitations of this study include its inherent selection bias given that only videos in the English language were included, which reduces the generalizability of our results to videos produced in other languages and originating from different geographical regions. In addition, our search was limited to YouTube. Other social media platforms such as Facebook, TikTok, Instagram, and Twitter were not included in this study, although these remain a target for future research due to high use as well.

### Conclusions

There is a significant degree of variation in the quality of health-related YouTube videos on the gut microbiome. Both the channel type and the presence of financial intent were significant factors in the quality, reliability, and transparency of the information provided. There is little correlation between viewership and information quality, reflecting a mismatch in public engagement and discernment of good-quality health advice from misinformation. This calls for greater scrutiny of health-related information provided on social media platforms. Further work should aim to impose more stringent regulations as well as policies and educational resources to ensure accurate and reliable information is accessible in a transparent manner with the interests of the general public in focus.

## Abbreviations

HONcode: Health on the Net Code of Conduct
UK: United Kingdom
